# A sneaky vertebra during a right inferior pulmonary vein laser ablation

**DOI:** 10.1016/j.hrcr.2021.07.003

**Published:** 2021-07-23

**Authors:** Hideyuki Aoki, Yuichi Hori, Reiko Fukuda, Shiro Nakahara

**Affiliations:** Department of Cardiology, Dokkyo Medical University Saitama Medical Center, Saitama, Japan

**Keywords:** Atrial fibrillation, Compression, Endoscopy, Laser ablation, Vertebra

## Introduction

Visually guided laser ablation (VGLA) in atrial fibrillation (AF) patients is performed by the combined use of endoscopy and provides us the exact structure of the pulmonary vein (PV) antrum. The ablated lesions by laser energy are visually detectable, which assists us in a precise point-by-point ablation creating a durable isolation line on the PV antrum.

Contact between the left atrium (LA) and extra-PV structures has been reported.^1^^,^^2^ However, these contacts are mainly demonstrated by static images, and whether they influence the development or treatment of AF is still unknown. We report a case of an AF patient who underwent a laser balloon ablation and in whom the contact between the right inferior PV (RIPV) antrum and vertebra was actually visualized by the endoscope.

**Key Teaching Points**

• The compression from the vertebrae on the right inferior pulmonary vein (RIPV) antrum can be visualized through endoscopy.
• The compression area from the vertebra moves correspondingly with the respiratory movements.
• The confirmation of a moving vertebra on the RIPV antrum is rare, and how this phenomenon affects the outcome of the ablation with the available ablation system needs further study.

## Case report

**Figure 1:**
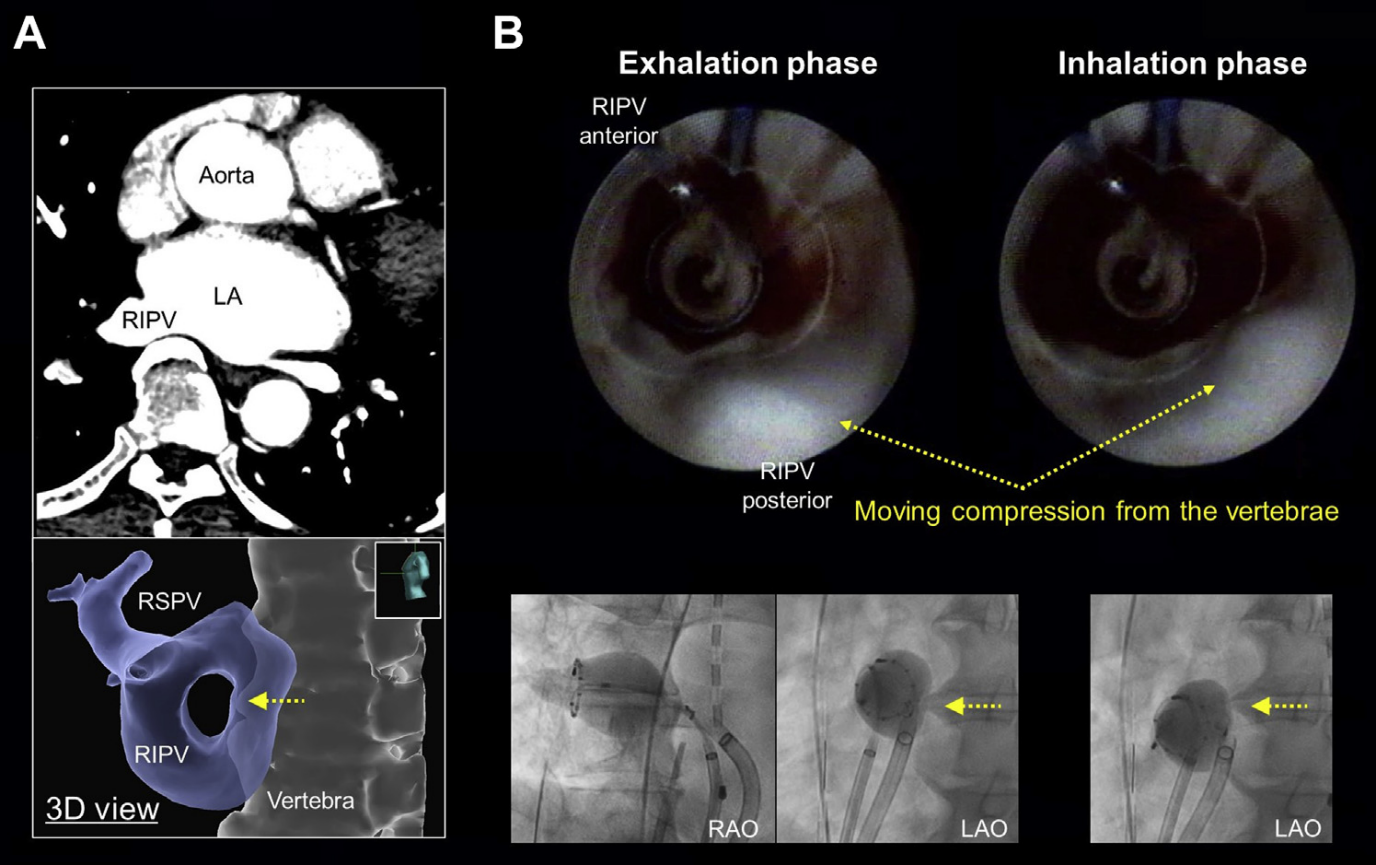
**A:** Enhanced computed tomography demonstrating strong contact from the vertebra (top panel) and 3-D view of the vertebra compressing the right inferior pulmonary vein (RIPV) antrum. **B:** Endoscopic view of the RIPV antrum is shown in top panel. The compression area from the vertebra moves corresponding to the respiratory movements. The bottom panel shows the fluoroscopic image of the laser balloon and vertebra during respiratory movements. The compression on the posterior region of the RIPV antrum was stronger on the inferior side (exhalation phase) than superior side (inhalation phase). LA = left atrium; LAO = left anterior oblique; RAO = right anterior oblique; RSPV = right superior pulmonary vein.

A 70-year-old man underwent a VGLA owing to paroxysmal AF. In the transthoracic echocardiography study, the LA diameter was 39 mm and no valvular disease was identified. Contrast-enhanced computed tomography was obtained before the ablation to confirm the anatomical information of the LA and PVs. The LA volume was 137 mL and the 4 PVs were all individual. An osteophyte was observed on the vertebra near the RIPV and strong contact between the RIPV antrum and vertebra was suggested (Figure 1A). The patient had no history of spinal deformities, compression fractures, or other spinal surgeries.

**Figure 2:**
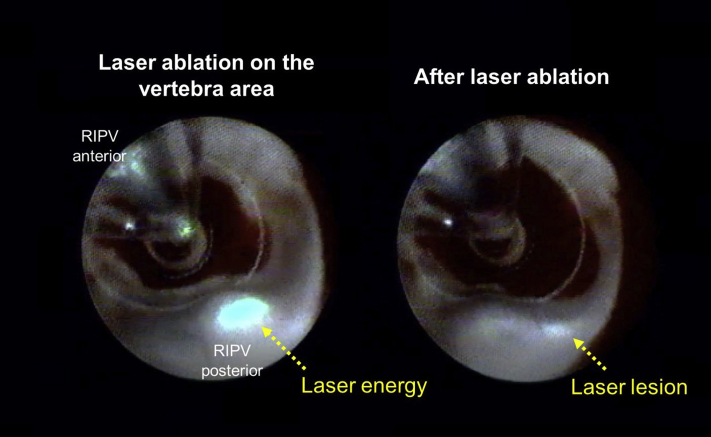
Laser ablation in the compression area with a moving vertebra. The movie is included as supplementary material. RIPV = right inferior pulmonary vein.

A first-generation VGLA catheter (HeartLight; CardioFocus, Marlborough, MA) was used for ablation and a mapping catheter (EPstar Libero; Japan Lifeline, Tokyo, Japan) was also placed into the distal PV for a real-time assessment of the PV isolation (Figure 1B, bottom panel). The patient was sedated with dexmedetomidine 0.5 μg/kg/h and pentazocine hydrochloride 30 mg. The left superior, left inferior, and right superior PVs were isolated successively by the laser ablation. Both the VGLA catheter and mapping catheter were placed at the RIPV antrum and the balloon was inflated to obtain a complete occlusion. The RIPV antrum was visualized through the endoscope and the compression from the vertebra was creating a bump on the posterior region (Figure 1B). The vertebra was moving correspondingly with the respiratory movements just at the RIPV antrum where the isolation is usually performed. The laser energy was delivered to the vertebra area, and the formation of a lesion was confirmed through the endoscopic view (Figure 2, Supplemental Movie). There was no increase in the esophageal temperature during the RIPV ablation and the isolation by the laser ablation system was accomplished with no complications. The patient has been free from any AF recurrence during 6 months of follow-up.

## Discussion

To the best of our knowledge, our image is the first to demonstrate real compression from the vertebra on the RIPV antrum. This compression area moved correspondingly with the respiratory movements. Further, the large movement of the target myocardium during the laser ablation was expected to affect the formation of a highly durable ablation lesion.

Regarding ablation using a radiofrequency catheter, creating a clean linear lesion on this moving vertebra area may be difficult from the point of maintaining sustainable contact. In contrast, the cryoballoon ablation system may be less affected, since the stability will be obtained by a complete and stable occlusion. In our institute, compression from the vertebra was observed in only 2 cases (2.8%) among 70 consecutive patients that underwent laser ablation. The compression between the RIPV and vertebra would be associated with anatomical factors, but its occurrence rate seemed to be low. Therefore, whether this phenomenon affects the clinical outcome of ablation with the current available ablation systems needs further study.

## Conclusion

The compression from the vertebra on the RIPV antrum was visualized in the endoscopic view during the VGLA. The compressed area moved around the posterior region of the RIPV antrum and corresponded to the respiratory movements. This movement may cause the RIPV antrum to be a challenging area for creating a durable lesion with some ablation systems.

## Appendix Supplementary data

Supplemental Video 1 Endoscopic view of the RIPV antrum during the laser ablation. The compression area from the vertebra moves along with the respiratory movements. The laser ablation lesion in the compression area was able to be confirmed through the endoscope.
