# Supplementary material for: A sneaky vertebra during a right inferior pulmonary vein laser ablation
Source: HeartRhythm Case Rep. 2021 Jul 23;7(9):637–9. doi: 10.1016/j.hrcr.2021.07.003 (PMC8441200; doi:10.1016/j.hrcr.2021.07.003)

**Supplementary material**

**Movie Legend**

Endoscopic view of the RIPV antrum during the laser ablation. The compression area from the vertebra moves along with the respiratory movements. The laser ablation lesion in the compression area was able to be confirmed through the endoscope.


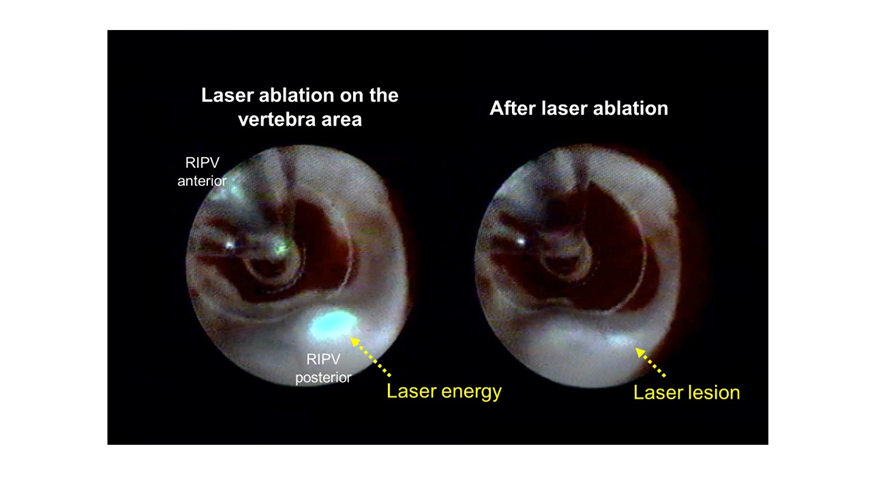

Supplement: Supplementary Material [file mmc2.docx]
